# An End-to-End Inclination State Monitoring Method for Collaborative Robotic Drilling Based on Resnet Neural Network

**DOI:** 10.3390/s24041095

**Published:** 2024-02-07

**Authors:** Lu Qian, Peifeng Liu, Hao Lu, Jian Shi, Xingwei Zhao

**Affiliations:** 1 School of Transportation and Logistics Engineering, Wuhan University of Technology, Wuhan 430062, China; qianlu@whut.edu.cn; 2 State Key Laboratory of Digital Manufacturing Equipment and Technology, Huazhong University of Science and Technology, Wuhan 430074, China

**Keywords:** robotic drilling, process monitoring, inclination state detection, machining signal processing, deep residual network

## Abstract

The collaborative robot can complete various drilling tasks in complex processing environments thanks to the high flexibility, small size and high load ratio. However, the inherent weaknesses of low rigidity and variable rigidity in robots bring detrimental effects to surface quality and drilling efficiency. Effective online monitoring of the drilling quality is critical to achieve high performance robotic drilling. To this end, an end-to-end drilling-state monitoring framework is developed in this paper, where the drilling quality can be monitored through online-measured vibration signals. To evaluate the drilling effect, a Canny operator-based edge detection method is used to quantify the inclination state of robotic drilling, which provides the data labeling information. Then, a robotic drilling inclination state monitoring model is constructed based on the Resnet network to classify the drilling inclination states. With the aid of the training dataset labeled by different inclination states and the end-to-end training process, the relationship between the inclination states and vibration signals can be established. Finally, the proposed method is verified by collaborative robotic drilling experiments with different workpiece materials. The results show that the proposed method can effectively recognize the drilling inclination state with high accuracy for different workpiece materials, which demonstrates the effectiveness and applicability of this method.

## 1. Introduction

Benefitting from the advantages of high-motion flexibility, large work space, high efficiency and strong parallel coordination ability, robots can adapt to complex processing environments, which promote the application potential of robotic drilling technology in the areas of aerospace manufacturing, automobile, and energy [1]. However, compared with numerical control machine tools, robots suffer from the inherent weaknesses of low rigidity and variable rigidity due to the series joint structure and varied robot pose, which severely limit the practicality and realization of robotic drilling with high performance [2,3]. In the process of practical robotic drilling, the rotation of the drill bit may generate forced vibrations, which makes the holes drilled by a robot more prone to inclination, affecting both surface quality and drilling efficiency. Therefore, it is critical to develop an effective and accurate robotic drilling-state monitoring method to improve the efficiency and quality of robotic drilling.

To ensure the quality of robotic drilling, a variety of sensors and means are used for monitoring the drilling process, such as acoustic emission [4], force [5,6], vision [7], vibration [8,9,10], and so on. Among them, vibration provides several unique advantages due to collection convenience, massive data, and its sensitivity to the running state of a mechanical system, which makes it show better application potential in practical engineering applications [11,12]. In essence, the state monitoring of robotic drilling is a state-classification problem. There exist two different states that we care about in the actual robotic drilling process, namely, vertical drilling and inclined drilling, which generate different features in the signals. Through state classification methods, we can theoretically detect the state of the drilling process based on different features. In terms of signal classification for the state monitoring of a mechanical system, it can be classed into two categories, according to the literature, namely a signal processing method and data-driven method.

During the past decades, a large number of signal processing methods have been applied for quality monitoring and equipment condition monitoring, which can be divided into three main classes according to the fundamentals of these methods, namely, time-domain analysis, frequency-domain analysis and time-frequency-domain analysis. Such as time-domain-statistical parameters [13], fast Fourier transform analysis (FFT) [14], wavelet transform [15,16], Hilbert–Huang transform [17,18], variational mode decomposition (VMD) [19,20], sparse representation [21,22], etc. Qin et al. [23] proposed a chatter detection approach to recognize robotic drilling chatter effectively, based on the concentrated velocity synchronous linear chirplet transform. In [24,25], the authors presented a robotic drilling chatter monitoring method, which can timely identify chatter precisely by extracting weak features of chatter in the initial period of the chatter occurrence. These signal processing methods can effectively achieve noise reduction and highlight features of interest in the signals, which will be conducive to realize reliable and accurate condition monitoring further. However, because of the system dynamics and stiffness characteristics of the robot, the vibration signals generated show strong time-varying characteristics. In addition, the signal processing methods only extract the signal features but do not mirror the characteristics of the system generally. Consequently, the classification accuracy depends on expertise and human intervention when processing the time-varying signal [26].

For the purpose of timely and accurate condition monitoring of complex systems, such as robot processing systems, many scholars in recent years have focused on the data- driven methods, which mainly contain statistical analysis [27], machine learning [28,29,30], and deep learning [31,32]. In these methods, deep learning methods have achieved fruitful results in big data processing due to the powerful modeling and representation capabilities. Through building a deep model and capturing hidden features in the data, deep learning can describe the rich internal information of the data, and finally improve the accuracy of classification or prediction [33]. Therefore, due to the excellent classification capabilities, deep learning methods can extract vital information that we are concerned about from the original data and distinguish abnormal states from normal states automatically. Abu-Mahfouz [34] monitored the tool-wear condition of a twist drill through comparing several architectures of the multi-layer feed-forward neural network. In this study, five different drill wear conditions were detected and classified effectively. Lu et al. [35] investigated a health status recognition method to detect the running state of machines based on a stacked denoising autoencoder (SDA), which can be used for the noisy signals and fluctuant running conditions. In [36], a two-stage intelligent learning framework is investigated to identify the health status of mechanical equipment, including an unsupervised sparse filtering to learn features adaptively and softmax regression to automatically classify the mechanical health conditions.

Deep learning methods have obtained abundant achievements in the state monitoring of mechanical systems based on vibration signals. However, only limited investigations on the quality monitoring of robotic drilling based on deep learning have been conducted. The dynamic response of the robot is far more complex than that of the machine tool, and the processing quality is affected by multiple sources such as process parameters and motion accuracy, which increases the difficulty of extracting the implicit relationships between vibration and processing quality. In addition, most of the existing research pays more attention to the tool-wear conditions and chatter identification in the robotic drilling process, while drilling inclination state identification is neglected, which reflects the quality of robot processing more intuitively.

Inspired by the above observation, in this paper, an inclination state monitoring framework for robotic drilling is developed, where the drilling inclination state is quantified through contour identification and an end-to-end drilling inclination state monitoring model is constructed. The main contributions of this paper are summarized as follows.

(1) An end-to-end state monitoring scheme for robotic drilling is explored, applying edge detection integrated with Resnet neural network techniques, which have the characteristics of high diagnosis accuracy, low resource consumption, and high real-time performance. With the aid of deep learning, the proposed method requires no prior knowledge, thus reducing the bias caused by human involvement. It is more applicable for processing massive signals in the context of big data monitoring.
(2) The Canny operator-based edge detection method is adopted to identify the profiles of the drilled hole, based on which the inclination angle is obtained to represent the inclination state of drilling. It provides data labeling information for the monitoring model training.
(3) Two experimental cases with different kinds of workpiece materials are implemented to demonstrate the feasibility and applicability of the method. Online monitoring of the drilling inclination state of collaborative robot is realized in a certain inclination angle range, where the accuracy reaches 100% for both of the two different workpiece materials.

The remainder of this paper is organized as follows: The characteristics of collaborative robots and the drilling processes are briefly described in Section 2. Section 3 details the robotic drilling inclination state monitoring method. In Section 4, collaborative robotic drilling experiments are carried out to verify the proposed method. Finally, some conclusions are given in Section 5.

## 2. Characteristic of Collaborative Robot Drilling

### 2.1. Stiffness Analysis of Collaborative Robots

Due to the properties of light weight and easy operation, the collaborative robot can simulate the human arm to complete the task of hole making in a narrow space. However, the inherent weaknesses of low rigidity and variable rigidity in robots bring detrimental effects to surface quality and drilling efficiency. For our purpose, the stiffness characteristics and drilling process of collaborative robot are analyzed first.

For most serial robots, the deformation of the robot mainly comes from the transmission device, reduction device, and servo drive system when it is subjected to external forces. Therefore, it is normally assumed that the robot link is absolutely rigid in stiffness modeling, and a spring coefficient $k_{i}$ is used to describe the stiffness of each joint in driving system, including the transmission and reducing mechanism, which is described as follows:

(1) $$\tau_{i}=k_{i}d\theta_{i}$$

where $\tau_{i}$ represents the torque of joint, $\theta_{i}$ denotes the joint variable, and $d\theta_{i}$ represents the deformation of the joint variable $\theta_{i}$ under the torque of joint $\tau_{i}$.

For a six-joint series robot, if the stiffness of each joint is $k_{1}$, $k_{2}$, ⋯, $k_{6}$, then the joint stiffness matrix can be described as $K_{\theta }=diag(k_{1},k_{2},k_{3},k_{4},k_{5},k_{6})$, where diag(⋅) represents the diagonal matrix composed of the elements in brackets. Further, Equation (1) can be transformed into the matrix form as follows:

(2) $$\tau =K_{\theta }d\Theta$$

where $\tau ={\left[\tau_{1},\tau_{2},\tau_{3},\tau_{4},\tau_{5},\tau_{6}\right]}^{T}$ denotes the joint torque vector, and $d\Theta ={\left[d\theta_{1},d\theta_{2},d\theta_{3},d\theta_{4},d\theta_{5},d\theta_{6}\right]}^{T}$ denotes the joint angle differential vector, namely, the deformation of the joint.

According to the differential kinematics of the robot and the Jacobian definition of the force field, the mapping relationship between deformation of the joint dΘ and the end deformation dX can be given as follows:

(3) dX=JdΘ

Meanwhile, the mapping relationship between the joint torque τ and the end force F is described as follows:

(4) $$\tau =J^{T}F$$

In Equations (3) and (4), J denotes the Jacobian matrix of the robot, $dX={\left[dx,dy,dz,d\alpha ,d\beta ,d\gamma \right]}^{T}$ denotes the generalized deformation of the robot end, and $F={\left[F_{x},F_{y},F_{z},N_{x},N_{y},N_{z}\right]}^{T}$ is the generalized external force acting on the robot end.

Considering Equations (2) to (4), the mapping relationship between the deformation of the robot end dX and the external force F can be obtained, namely, the stiffness model of the robot is described as follows:

(5) F=KdX

where ***K*** represents the Cartesian stiffness matrix of the robot, which can be given through the joint stiffness matrix $K_{\theta }$ as follows:

(6) $$K=J^{-T}K_{\theta }J^{-1}$$

Due to the inverse operation of the Jacobian matrix ***J*** in Equation (6), the calculation errors are usually introduced in this process, especially near the singular configuration. To avoid this problem, Abele et al. [37] proposed a compliance model for robot stiffness, namely:

(7) dX=CF

where ***C*** is Cartesian flexibility matrix, it is given as follows:

(8) $$C=JK_{\theta }^{-1}J^{T}$$

Comparing Equations (8) with (6), it can be found that the flexibility matrix ***C*** is the inverse matrix of the Cartesian stiffness matrix ***K***. Since the calculation of ***C*** does not involve the inverse operation of the Jacobian matrix ***J***, the stability and precision of the solution are improved.

Considering that in the actual processing tasks, such as grinding and drilling, the robot is mainly subjected to cutting forces, and the magnitude of the rotational deformation under the non-cutting torque is ignorable compared to the translational deformation. Thus, Equation (7) can be further expressed as follows:

(9) $$\left[\begin{array}{c} dX_{t}\\ 0 \end{array}\right]=C\left[\begin{array}{c} F_{f}\\ 0 \end{array}\right]$$

where $dX_{t}={\left[dx,dy,dz\right]}^{T}$ represents the translational deformation of the end of the robot, and $F_{f}={\left[F_{x},F_{y},F_{z}\right]}^{T}$ denotes the external force on the end of the robot. Furthermore, the flexibility matrix can be expressed in blocks as follows:

(10) $$C=\left[\begin{array}{ll} C_{tt} & C_{tr}\\ C_{tr}^{T} & C_{rr} \end{array}\right]$$

where ***C****_tt_*, ***C****_tr_* and ***C****_rr_* are the translational flexibility matrix, rotational flexibility matrix, and coupling flexibility matrix, respectively, and their units are m/N, rad/(N·m) and rad/N.

It follows from Equations (9) and (10) that the mapping relationship between the deformation of the robot end $dX_{t}$ and the external force $F_{f}$ in the Cartesian space is given as follows:

(11) $$dX_{t}=C_{tt}F_{f}$$

The stiffness of the robot is influenced by the deceleration, transmission, and servo devices of each joint; thus, it is difficult to achieve the stiffness of the machine tool in structure. According to the above derivation process, it is evident that the translational flexibility matrix ***C****_tt_* is not a fixed value, but changes with different postures of the robot. Hence, it is obvious that the stiffness of the robot changes with the posture, that is, the robot has variable rigidity.

**Figure 1:**
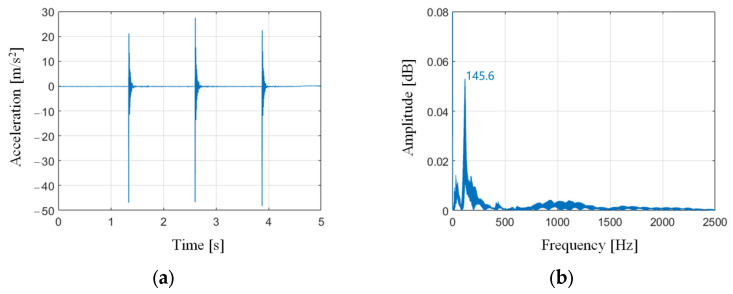
Vibration signal under impulse response tests, (**a**) time domain, (**b**) frequency domain.

To measure the stiffness of the robot, impulse response tests are conducted on the collaborative robot. An impulse signal is added at the end effector of the robot arm with a hammer, and the response vibration is measured by the acceleration sensor. Then, the critical eigen frequency and damping can be calculated as follows:

(12) $$\omega_{n}=\sqrt{k_{r}/m_{r}},C_{r}=2\sqrt{m_{r}k_{r}}$$

where $\omega_{n}$ is the eigen frequency, $m_{r}$ denotes the vibrated mass, $k_{r}$ represents the critical stiffness, $C_{r}$ is the critical damping. Figure 1 shows the vibration signal under the impulse response tests. It has a main frequency at 145.6 Hz. By choosing the mass of the end effector of the robot as the vibration mass, the stiffness of the robot is 4.2 × 10^4^ N/m, while the critical damping is 582.4 Ns/m.

**Figure 2:**
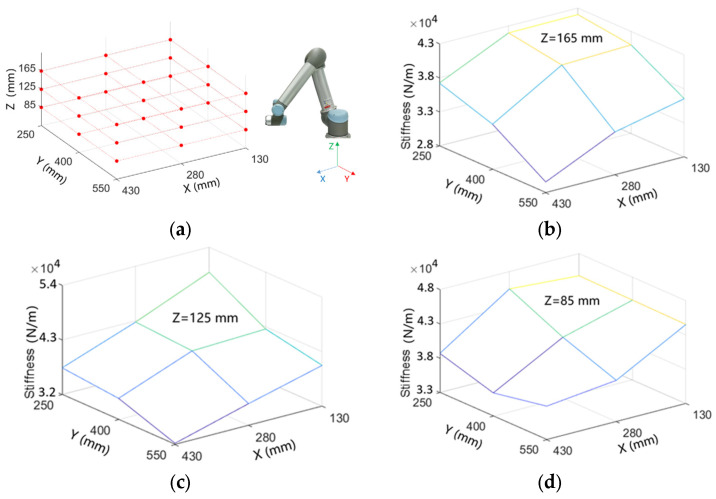
Stiffness map of collaborative robot, (**a**) the measurement points, (**b**–**d**) the stiffness map under different postures.

Then, 27 measurement points are chosen in the workspace of the robot, which covers the main workpiece of the robot. Its stiffness map can be calculated by measuring the impulse response of the robot, as shown in Figure 2. The stiffness of the collaborative robot under different postures is within 3~4.5 × 10^4^ N/m, which is much lower than the stiffness of machining tools.

### 2.2. The Drilling Process of Collaborative Robot

**Figure 3:**
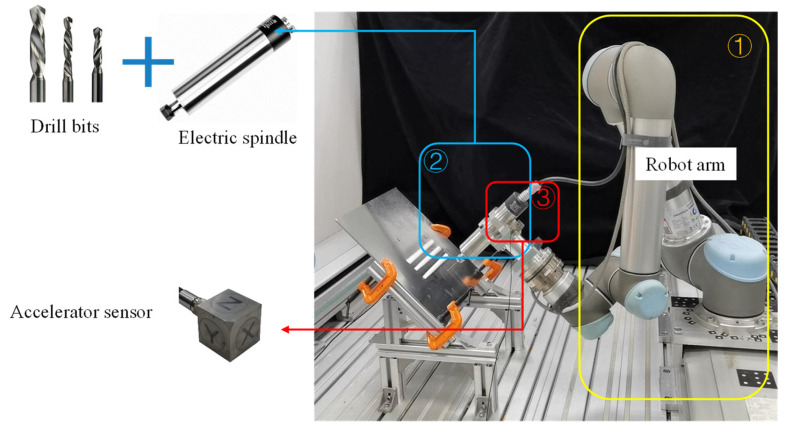
Drilling scenario of collaborative robot.

**Figure 4:**
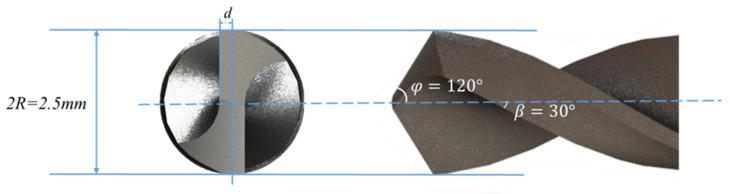
The geometry of the drill.

**Figure 5:**
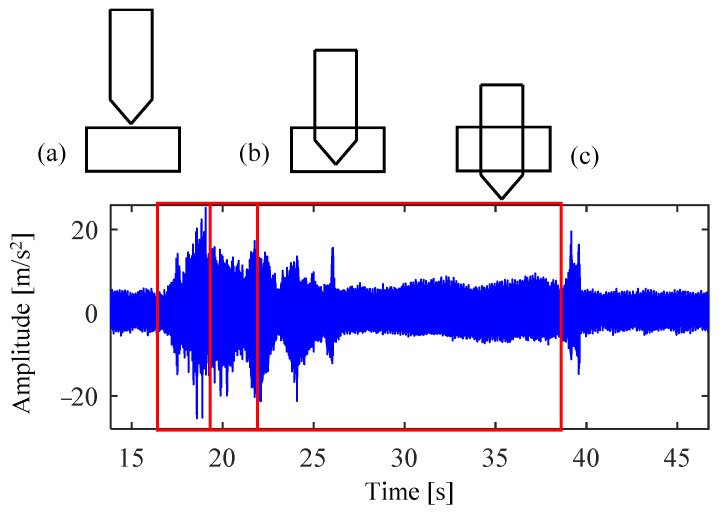
Schematic diagram of the robot drilling process, (**a**) initial drilling, (**b**) steady drilling, (**c**) drilling through.

Drilling generally refers to the operation of machining a hole in a workpiece using a drill bit. The robot drilling is to install the drill bit at the end of the serial manipulator and rely on the joint movement of the manipulator to realize the drilling operation at the preset point. Figure 3 shows a drilling scenario of collaborative robot. Figure 4 gives the geometry of the twist drill used in this study, where the diameter is 2.5 mm, and chisel edge angle and helix angle are 120° and 30°, respectively. As shown in Figure 5, there exist three stages in the overall drilling process: (a) initial drilling stage, (b) steady drilling stage, (c) and drilling through stage. In the initial drilling stage, the drill bit enters into the workpiece until it entirely sinks in the workpiece. Then the drilling process enters a steady drilling stage, after the drill bit sinks in the workpiece until it touches the underside of the workpiece. In the drilling-through stage, the drill bit fully penetrates the underside of the workpiece, and the wall of hole completely touches the side wall of the drill bit. The three stages of the robot drilling process and the corresponding vibration signals are shown in Figure 4. Due to the probable unstable chatter, the signals of the initial drilling stage and drilling through stage show unsteady and unstable, it is better to choose the vibration data during the steady drilling process to recognize the drilling quality. As the trajectory is designed by the operator, the steady drilling process can be selected after 3 s when the robot contacts the workpiece.

Due to the weak rigidity of the robot, the workpiece will also affect the posture of the drill bit in the descent process of the drill bit. For industrial robots with relatively high rigidity, the feed trajectory of the robot can be approximated to the actual motion trajectory. But in the case of collaborative robots, there exists a certain deviation between the preset trajectory and the actual trajectory. Assuming that the external force from the workpiece is $F_{f}$, the real posture of the robot is given as follows:

(13) $$X_{t}=X_{r}+C_{tt}F_{f}$$

where $X_{r}$ denotes the preset trajectory, and $X_{t}$ represents the actual trajectory.

**Figure 6:**
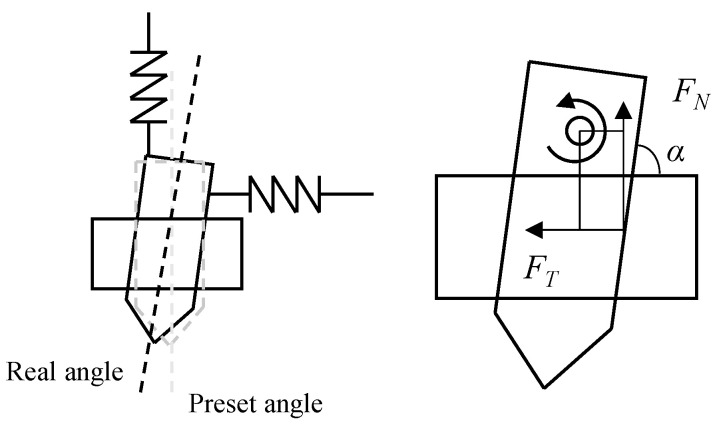
Force analysis of drilling process.

When the robot is inclined downward, the robot drill will receive an upward reactive force from the workpiece wall. There will be a certain deviation between the actually generated inclination angle and the preset inclination angle, as shown in Figure 6. Therefore, for the end-to-end training process, the real inclination angle should be used to evaluate the drilling quality but not the preset inclination angle.

## 3. End-to-End Robotic Drilling Inclination State Monitoring

**Figure 7:**
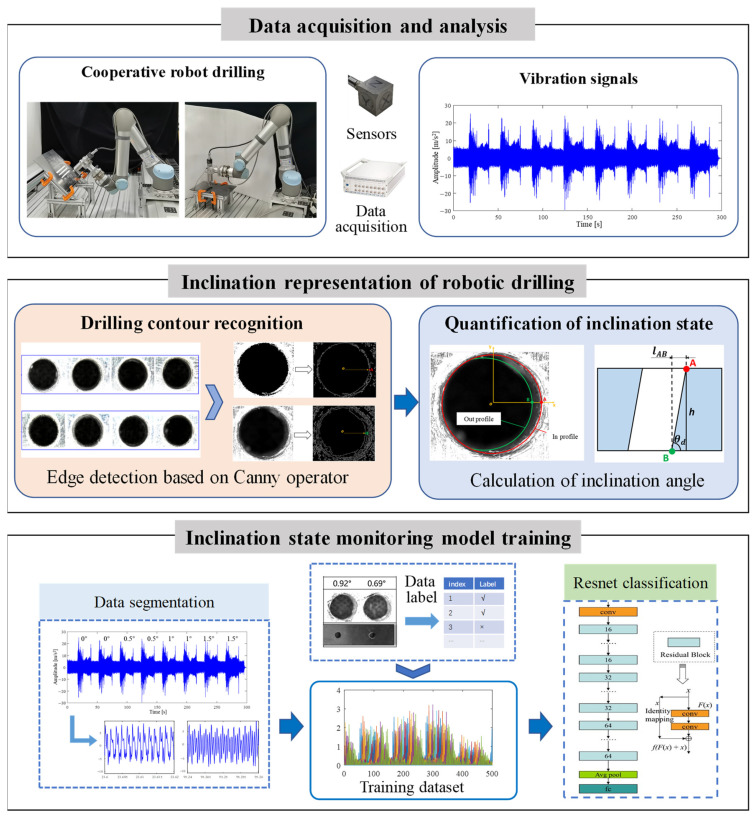
Framework of the robotic drilling inclination state monitoring method.

The framework of the proposed end-to-end robotic drilling inclination state monitoring method mainly includes three parts: 1. Date acquisition and analysis; 2. Inclination representation of robotic drilling; 3. Inclination state monitoring model training, as shown in Figure 7. First, a collaborative robot drilling processing platform is set up to acquire the vibration data of the robot drilling process under deferent conditions. Then, a Canny operator-based edge detection method is adopted to identify the profiles of the drilled hole to quantify the inclination state of the hole with the inclination angle, which contributes to data labeling for further state monitoring model training. Finally, an end-to-end robotic drilling inclination state monitoring model is trained, where the stationary vibration signals in the process of robot drilling is intercepted as the input signals, and the Resnet network is used for the drilling inclination state classification. After training, the inclination state of the drilled hole could be identified automatically by inputting the online vibration signals, and hence, the end-to-end robotic drilling inclination state monitoring is realized.

### 3.1. Vibration Data Acquisition and Analysis

In order to acquire the vibration data of the collaborative robot drilling process, a robot drilling processing platform is set up in this paper, as shown in Figure 3, which mainly includes the following modules: (1) trajectory execution module, (2) machining execution module, (3) data acquisition module. In what follows, the three modules are introduced in detail.

The trajectory execution module is a UR5 manipulator arm, which is responsible for executing the preset trajectory. The technical parameters of the manipulator arm are given in Table 1.

The machining execution of this system is completed by the motorized spindle installed at the end of the manipulator, and the drill bit can be switched and installed on the tool-holding end of the motorized spindle. The data acquisition module contains an acceleration sensor. The acceleration sensor is installed on the clamping tool of the motorized spindle close to the drill bit. The technical parameters of the acceleration sensor and data acquisition instrument are shown in Table 2.

**Figure 8:**
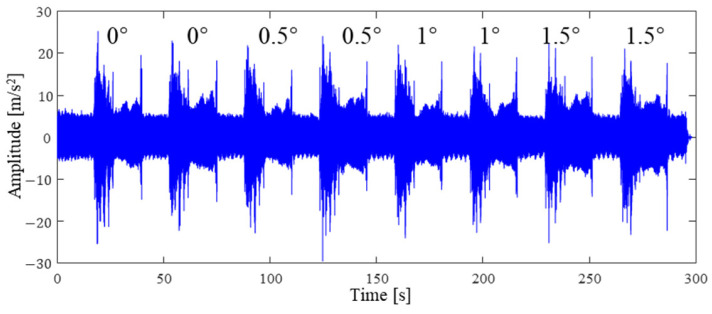
Measured vibration signal under different preset inclination angles.

**Figure 9:**
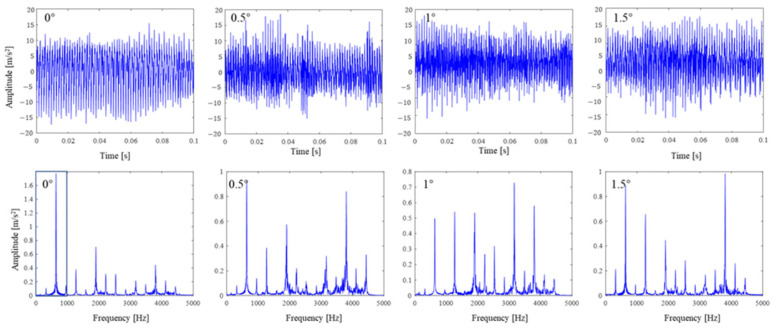
The frequency analysis of the vibration signal under different preset inclination angles.

To acquire vibration data under different inclination states, different preset angles, from 0 degree to 1.5 degree, are set. The actual inclination angle will not be consistent with the preset angles because of the robot’s low rigidity. The vibration signals are recorded by the data acquisition instrument, as shown in Figure 8. It is hard to identify the preset inclination angle from the signals in the time or frequency domain, since many frequency peaks are excited during drilling, as shown in Figure 9.

### 3.2. Inclination Detection of Robotic Drilling

A visual system is used to measure the real inclination angle during robotic drilling. In order to realize the quantitative representation of the inclination state of robotic drilling, an edge detection method is adopted to identify the profiles of the hole drilled to quantitatively evaluate the inclination state of robotic drilling.

We use an edge detection method to identify the profiles of the hole on the upper surface and lower surface of the workpiece. Then we can obtain the geometric position relationship between the profiles of the hole on the upper and lower surface, based on which the inclination angle of the hole can be further acquired by calculations. Edge detection is to construct an edge detection operator based on a certain neighborhood of pixels in the image. General edge detection operators contain a Roberts operator, Sobel operator, Prewitt operator, Laplace operator and Canny operator. According to the criterion, it can be concluded that the Canny edge detector is the optimal approximation operator for the product of the signal-to-noise ratio and positioning by using functional derivation. In addition, in the Canny operator edge detection we applied the closing operation (dilation followed by erosion) for morphological filtering, which makes the edge around the hole smoother and removes the influence of noise caused by cutting chips to some extent. Then hough circle transform is used to find reliable boundaries. The procedure of the Canny operator edge detection are as follows:

(1) Gaussian smoothing

If the input image is expressed by *f*(*i*,*j*), we can obtain the convolution of the image and the Gaussian smoothing filter through the separable filtering method, then a smoothed data matrix can be given as follows:

(14) S(i,j)=G(i,j;σ)×f(i,j)

where σ is the standard deviation of the Gaussian function, which controls the smoothness.

(2) The calculation of gradient magnitude and direction angle

For the gradient of the smoothed data matrix S(i,j), the matrices P(i,j), and Q(i,j) of partial derivatives in the *i* and *j* directions can be calculated by using the 2 × 2 first-order finite difference approximation.

(15) P(i,j)≈(S(i+1,j)−S(i,j)+S(i+1,j+1)−S(i,j+1))/2Q(i,j)≈(S(i,j+1)−S(i,j)+S(i+1,j+1)−S(i+1,j))/2

Then, finite differences are averaged within this 2 × 2 square in order to calculate the partial derivative gradients of *i* and *j* at the same point in the image. Through the coordinate transformation formula from the Cartesian coordinates to polar coordinates, the magnitude and direction angle is given as follows:

(16) $$M(i,j)=\sqrt{P{\left(i,j\right)}^{2}+Q{\left(i,j\right)}^{2}}\;\theta (i,j)=\arctan(Q(i,j)/P(i,j))$$

where M(i,j) reflects the edge strength of the image and θ(i,j) represents the direction of the edge.

(3) Non-maxima suppression

The larger the value of the image magnitude matrix M(i,j), the greater the corresponding image gradient value. But it is insufficient to identify the edge, since here the problem of fast image transformation is just transformed into the problem of finding the local maximum value of the magnitude matrix M(i,j). In order to determine the edge, the ridge band in the magnitude image must be refined, that is, only the point with the largest local change in magnitude is reserved, which is called Non-maxima suppression (NMS).

Through suppressing the magnitudes of all non-ridge peaks on the gradient line, NMS refines the gradient magnitude ridges in M(i,j). This algorithm reduces the variation range of the gradient angle θ(i,j) to one of the four sectors of the circle, namely, the normalization of the direction angle:

(17) ς[i,j]=Sector(θ[i,j])

Labeling the four sectors from 0 to 3, corresponding to the four possible combinations in the 3 × 3 neighborhood, means any point that passes through the center of the neighborhood must pass through one of the sectors. The circle partition in the possible direction of the gradient line is marked with degrees, and the algorithm uses a 3 × 3 neighborhood over all points in the magnitude matrix M(i,j). The central pixel amplitude M(i,j) of the neighborhood at each point is compared with the two elements along the gradient line, which is given by the sector value ς(i,j) at the point of the neighborhood. If the amplitude M(i,j) at the center point of the neighborhood is not greater than the amplitude of two adjacent points along the gradient line, then we assign zero to M(i,j). This process refines the wide ridge band to only one pixel. During the process of *NMS*, the ridge height values are preserved. Assume that the *NMS* process is described as follows:

(18) N(i,j)=NMS(M(i,j),ς(i,j))

where non-zero values of N(i,j) are associated with the contrast ratio at steep changes in image intensity. In spite of the image smooth in the beginning of edge detection, N(i,j) still involves certain false edges due to noise and texture. In practical application, the contrast of false edges is generally small, which can be filtered by setting a threshold.

(4) Double threshold detection

In order to solve the problem of false edges, Canny proposed a double-threshold method. Through the cumulative statistical histogram, one obtains a high threshold and a low threshold. If the response of the image signal is larger than the high threshold, then it can be identified as the edge. While it is lower than the low threshold, it is not identified as the edge. If it lies between the high threshold and the low threshold, it depends on whether its eight adjacent pixels have an edge larger than the high threshold. If this exists, then it is an edge, otherwise it is not an edge.

**Figure 10:**
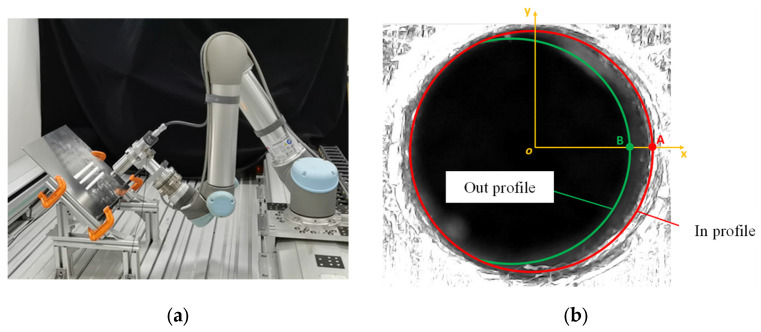
The image of the hole taken perpendicular to the workpiece surface and calculation of the inclination of the hole. (**a**) UR5 robotic arm from Universal Robots in Odense, Denmark, (**b**) calculation of the inclination of the hole.

A robot visual inspection system is given in Figure 10a, while Figure 10b shows the measured image of the hole taken vertically to the workpiece. In theory, the profiles of drilling in and drilling out can be obtained by edge detection and circle fitting, and then the farthest distance between the two circles can be found to serve as the displacement of the hole. However, there exists a certain degree of blurring in the drilling out profile due to the unsmooth edge from drilling into metal and the problem of focal length and light, which increases the difficulty of the circle fitting of the drilling out. Therefore, when the drilling dataset is built through experiments, the inclination direction of the hole is fixed by presetting the inclination angle, which ensures that the maximum distance between the two profiles is at the position y=0(x>0) of the image. In this way, only the positions of points A and B need to be found, as shown in Figure 10b.

**Figure 11:**
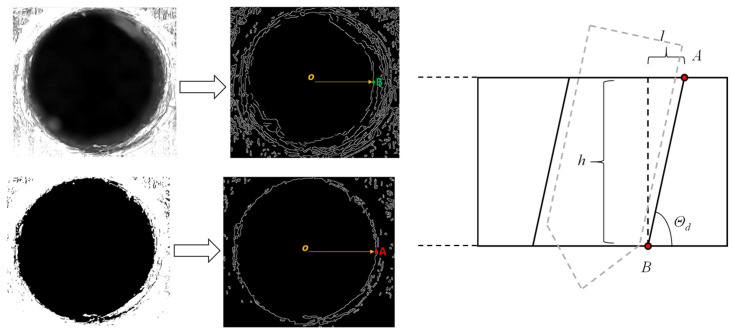
Edge detection based on Canny operator.

To obtain the inclination angle of the hole, the Canny operator edge detection is first carried out on the original image to obtain the drilling out profile, and the position of point B can be found by traversing from the image center *o* to the right, as shown in Figure 11. Then, the binarization of the original image is performed to eliminate the hole wall area with low pixel value, based on which the Canny operator edge detection is carried out to obtain the drilling in profile. At this time, the position of point A can be found by traversing from the center of the image *o* to the right. Finally, if the horizontal distance from point A to point B is $l_{AB}$, and the thickness of the workpiece is *h*, then the inclination of the hole can be given as follows:

(19) $$\theta_{d}=\arctan(\frac{h}{l_{AB}})$$

### 3.3. Inclination State Monitoring Model Training

Since the inclination angle of the collaborative robot drilling is related to the actual drilling conditions, it is necessary to detect the drilling state in real time. Especially when the number of holes reaches the tens of thousands, this real-time state detection becomes particularly important. For practical collaborative robot drilling, the actual inclination angle will not agree with the preset inclination angle. To this end, an end-to-end training framework is proposed in this paper, where the relationship between the actual inclination states and the vibration signals is trained by using the vibration data and the actual inclination states as the training set.

**Figure 12:**
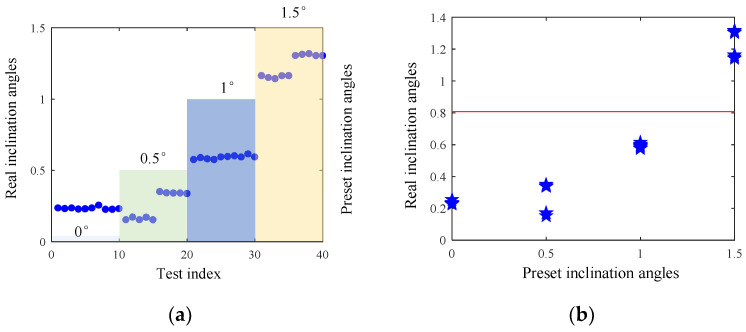
The preset angles and the corresponding actual inclination angle of collaborative robot drilling, (**a**) differences between the preset and actual inclination angle, (**b**) the classification boundary between acceptable and unacceptable angle.

We use collaborative robots to drill 40 holes, whose working conditions are shown in Table 3. We drill 10 holes with each preset angle, where the feeding speed of five holes is 0.5 mm/s and the other five holes is with a feeding speed of 1 mm/s. Figure 12 shows the different preset angles and the corresponding actual inclination angle of collaborative robot drilling. When the preset angles are set as 0° and 0.5°, the actual inclination angle is about 0.3°. It follows that, due to the mechanical deformation of the robot, the slight change in preset angle shows little influence on the actual inclination angle. In addition, robots are difficult in achieving attitude adjustment with a high precision due to the limited control accuracy of collaborative robots. When preset angles are 1°, the actual inclination angle is about 0.6°. While preset angles are set as 1.5°, the actual inclination angle is between 1.1°and 1.3°. It can be seen that the actual inclination changes obviously when the preset inclination changes from 1° to 1.5°. Therefore, in the end-to-end training process, the acceptable angle is set as the real inclination angle lower than 0.8 degree (the preset angle lower than 1 degree), while the unacceptable angle is set as the real inclination angle larger than 0.8 degree (the preset angle larger than 1 degree), as shown in Figure 12b. The data groups 1–30 are set as the normal group, and the groups 31–40 are set as the abnormal group. Then, a Resnet classification network is used to distinguish the normal and abnormal groups.

**Figure 13:**
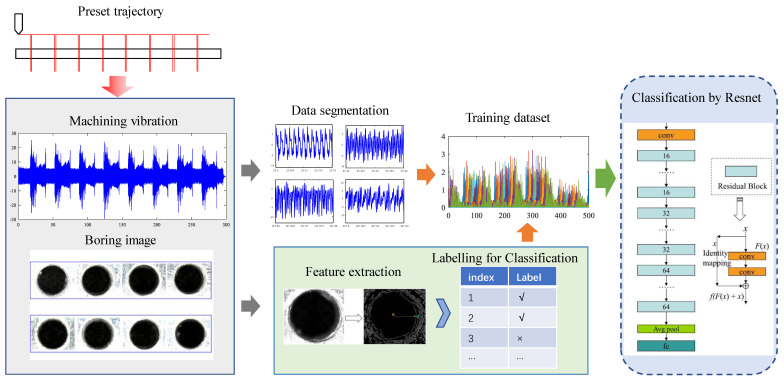
End-to-end classifier training method for robotic drilling.

Figure 13 shows an end-to-end classifier training framework for robotic drilling based on vibration signals, where Resnet is used for the drilling inclination state classification. In order to obtain a reasonable training set, the stationary vibration data in the process of robot drilling are taken as the input signals, which is labeled by the drilling quality. The data are divided into normal and abnormal categories with the real angle of 1° as the boundary, which is then fed into the Resnet network to train a suitable robot drilling-state classifier.

The Resnet network architecture comprises an input layer, a convolutional layer, four residual shrinkage units (ResUnit), a batch normalization layer (BN), a ReLU activation function layer, a global average pooling layer, and a full connection layer (GAP). The convolution layer employs a convolutional kernel with a kernel size of eight. Each residual shrinkage unit is composed of kernels with varying sizes. The subsequent BN, ReLU, and Pooling layers can maintain their default parameters. The final FC layer utilizes the softmax activation function. In addition, the batch size will be set to 256. Table 4 shows the structure parameters of Resnet.

**Figure 14:**
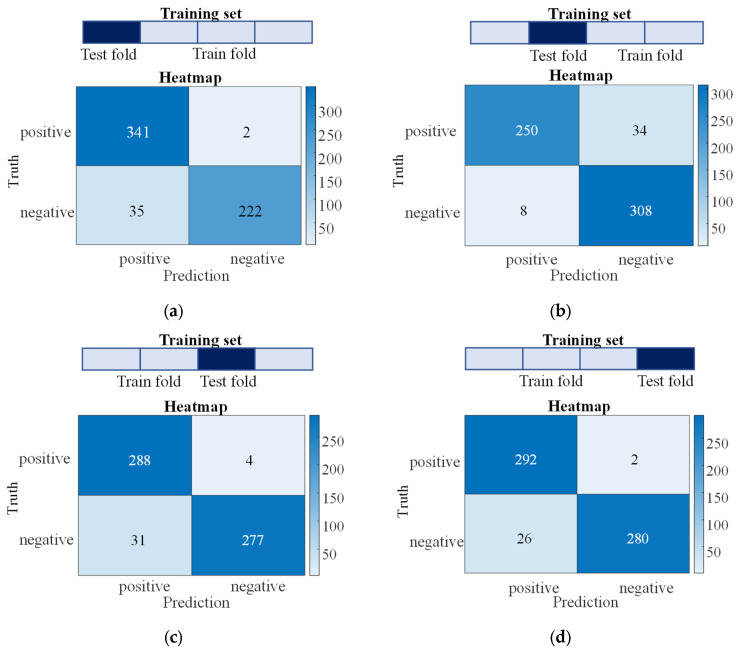
Confusion matrix of four different datasets for testing, (**a**–**d**) for different selected training data.

The whole data comes from a 40 group test experiment. A total of 60 data slots are extracted from each test data. Thus, 2400 group training data are obtained. In those data, 1800 group data is used as training data, while 600 group data are treated as test data. The inclined holes are set to be positive samples for the neural network model and the vertical holes to be negative samples, where then we can then construct the confusion matrix for this binary classification problem, as shown in Figure 14. The accuracy performed on the training set can reach more than 95%. The dataset is separated into different training folds and the test are folded multiple times for cross validation. Specifically, the training dataset is equally divided into four folds, and one of them is taken as the validation fold, and the remaining three parts are taken as the training fold, so as to obtain better bias estimation to verify the stability and effectiveness of the model. The accuracy on the test set can all reach more than 93%. After training, the accuracy performance of the test data is about 95%. We can see from the confusion matrix that the model’s misclassifications mostly involve mistakenly identifying positive samples as negative, while almost all negative samples are correctly detected. which is acceptable for ensuring processing quality in practical scenarios. The results indicate that the training process is efficient for the drilling state monitoring.

## 4. Experimental Verification

**Figure 15:**
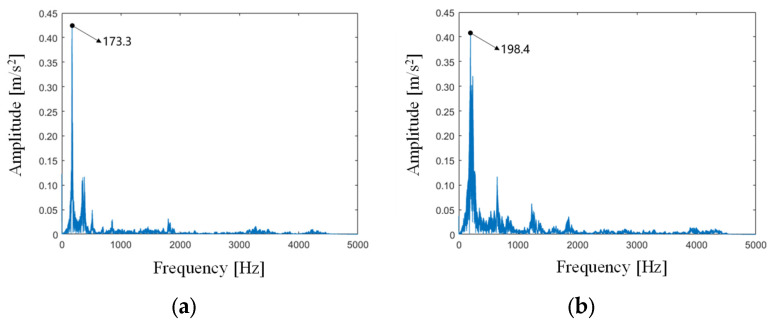
The natural frequencies, (**a**) aviation aluminum, (**b**) carbon fiber composite.

**Figure 16:**
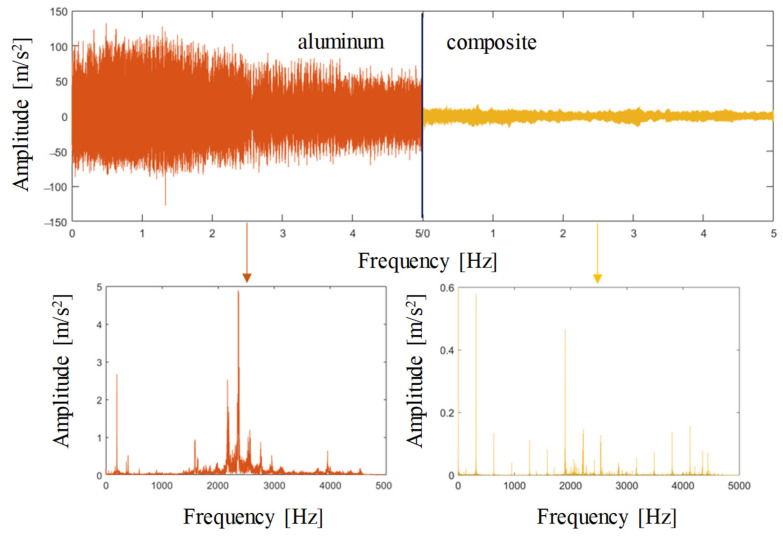
Comparison of vibration signals between aviation aluminum and carbon fiber composite.

In this section, the collaborative robotic drilling experiments are carried out to testify the proposed robotic drilling-state monitoring method, where two kinds of materials were tested to demonstrate feasibility and applicability, namely aviation aluminum and carbon fiber composite. Due to the differences in structure and material properties between the two materials, training data were prepared separately for each material. Figure 15 shows the natural frequencies of the workpieces of two materials measured by the hammer method. The static stiffness and critical damping coefficient are calculated based on Equation (12). Here the mass of the aluminum plate and composites board are 0.636 kg and 0.394 kg, respectively, and we can calculate that the stiffness of the aluminum plate is 19,101 N/m, the damping coefficient is 220 N·s/m, the stiffness of composite board is 15,509 N/m, and the damping coefficient is 156 N·s/m. The comparison of vibration signals between aviation aluminum and carbon fiber composite is shown in Figure 16. It is indicated that there exist obvious differences in the modal parameters and vibration signals between the two materials.

A. Drilling-state monitoring for aviation aluminum materials

**Figure 17:**
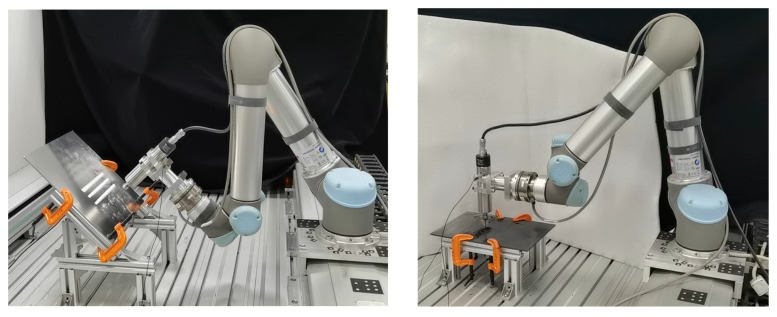
Cooperative robot drilling scenarios of aviation aluminum and composite materials, where the robotic arm is UR5 from Universal Robots in Odense, Denmark.

Figure 17 shows the cooperative robot drilling scenarios of aviation aluminum and composite materials. The training set is formed by 16 groups of robot drilling data, where the preset angles are 0°, 0.5°, 1.0°, and 1.5°, respectively. A total of four holes were drilled at each preset angle. We set the feed speed as 0.5 mm/s and the drilling speed as 350 r/s. The inclination state monitoring model is trained according to the training process in Section 3.3, where the actual critical angle between vertical and inclination angle is 0.8 degree.

**Figure 18:**
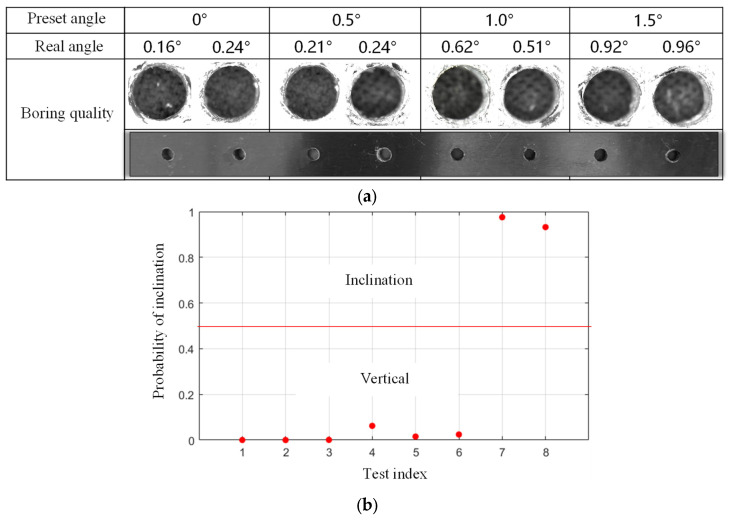
Drilling effect of aviation aluminum by cooperative robot, (**a**) the preset and real angles, (**b**) detection results of robotic drilling inclination state for aluminum plate.

After the training, the robot drills eight holes at the testing set. Each preset angle has two holes, as shown in Figure 18a. Based on the edge detection method, the actual inclination angles of the holes are, respectively, identified as 0.16°, 0.24°, 0.21°, 0.24°, 0.62°, 0.51°, 0.92°, 0.96°. The results of robotic drilling inclination state detection for aviation aluminum plate are given in Figure 18b. It can be seen that when the actual inclination angles of the holes on the aluminum plate is 0.92°and 0. 96°, it is identified as an inclined drilling state by applying the proposed robotic drilling inclination detection method. It follows that the proposed method can separate the inclined drilling and non-inclined drilling effectively.

B. Drilling-state monitoring for carbon fiber composite materials

To demonstrate the applicability of the proposed method, a cooperative robot drilling experiment is also carried out in composite materials. A total of 16 groups of robot drilling data are used as the training set and 8 groups of robot drilling data are used as the testing set. The drilling speed and the feed speed are set as the same as the experiment with aviation aluminum. We set the actual critical angle between vertical and inclination states as 0.5 degree.

**Figure 19:**
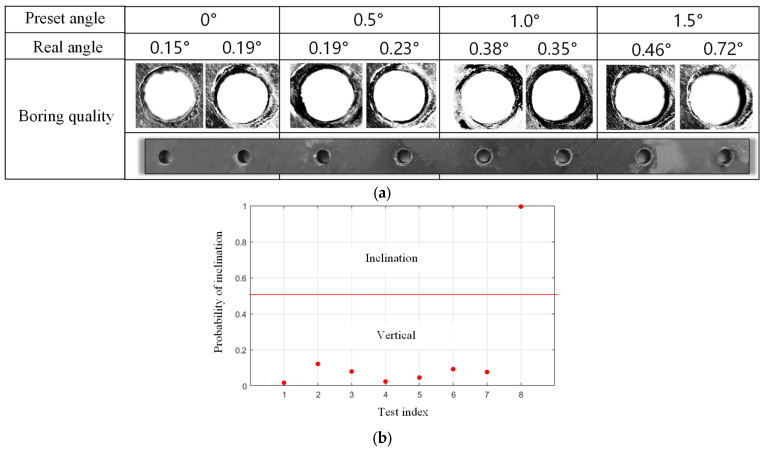
Drilling effect of composite materials by cooperative robot, (**a**) the preset and real angles, (**b**) detection results of robotic drilling inclination state for composite plate.

The eight holes are arranged in a straight line and two holes are drilled at each preset angle, which are 0°, 0.5°, 1.0°, and 1.5°, as shown in Figure 19a. The actual inclination angles of the holes are identified based on the edge detection method as 0.15°, 0.19°, 0.19°, 0.23°, 0.38°, 0.35°, 0.46°, 0.72°, respectively. The detection results of robotic drilling inclination state of composite materials are given in Figure 19b. It can be seen that when the actual inclination angle of the hole on the composite plate is 0.72°, it is identified as inclined drilling state. This result shows that the proposed method is also suitable for composite materials, and the identification resolution is similar to that of aluminum plate drilling, which demonstrates the effectiveness and extensibility of this method.

With regard to the delay in the real-time monitoring, the drilling inclination state of the hole can only be identified after each hole is drilled, where stable drilling signals acquisition and data processing based on Resnet classifier consume 0.028 ms and 0.054 ms, respectively, on a personal computer (CPU: i7 7700k, GPU: GTX 1060). Thus, the total time delay of the method reaches as low as 0.082 ms after each hole is drilled, which demonstrates the real-time performance of the algorithm.

C. Discussions

The actual inclination angle when drilling is slightly different from the preset inclination angle due to the weak rigidity and variable rigidity of robot. In order to obtain more accurate results, the actual inclination of each test hole is calculated by the contour recognition method. In general, because the drill bit is constrained by the hole wall, the actual inclination angles are smaller than the preset angles overall, as we can see in Figure 17a and Figure 18a. However, it is still largely related to the magnitude of the preset inclination angle. According to requirements of various drilling scenarios, the definition of actual inclination state, namely inclination angle range, can be modified in practical application. This work realizes online monitoring for drilling inclination state of collaborative robot in a certain inclination angle range, where the accuracy reaches 100% in the experiments. Compared to the methods that rely on laser tracking detection and traditional vibration signal analysis, this study could directly capture discriminative information of the drilling quality through establishing the matching relationship between vibration and inclination state, requiring no prior knowledge and reducing the bias caused by human involvement. Meanwhile, it possesses low-resource consumption, high real-time performance, and the deep learning-based classification methods offer higher credibility than traditional methods that require human inference. From a holistic perspective, the proposed method demonstrates scientific rigor and practical significance.

## 5. Conclusions

In this paper, an end-to-end inclination state monitoring method is developed for robotic drilling process. For our purpose, the drilling process of the collaborative robot is analyzed first. To quantify the inclination state of robotic drilling, the Canny operator-based edge detection method is used for drilling contour recognition, based on which the inclination angle is obtained to represent the inclination state of drilling. In addition, the robotic drilling inclination state monitoring model is constructed based on Resnet network. According to different inclination states represented by the inclination angle, the training dataset is constructed and the Resnet classifier is trained for the drilling-state classification. The proposed method is testified by collaborative robotic drilling experiments. In the cases of the two different workpiece materials, the inclined drilling and non-inclined drilling states were 100% identified by the proposed method, which verifies the effectiveness and applicability of the proposed method.

This paper has specifically explored an online monitoring scheme for robotic drilling with the characteristics of easy implementation in hardware, low-resource consumption, and monitoring delay. The vibration signals are easy to collect with the acceleration sensor installed near the motorized spindle, and the total monitoring delay is as low as 0.082 ms, after each hole is drilled. It is worth pointing out that the classification errors could be further reduced along with the increase in the number of the data.

There are some issues that can be considered in future work: (1) In the actual processing scenarios, process technology, working conditions and processing materials change frequently. Therefore, the adaptability of inclination state monitoring method cross working conditions and processing materials can be further studied through deep transfer learning in future work. (2) Based on the consideration that it takes much time and effort to obtain enough data in practical applications, thus inclination state monitoring method with a model-aided data augmentation technique can be further developed.

## Figures and Tables

**Table 1:** Main technical parameters of UR5 manipulator.

Technical Index	Payload (kg)	Effective Working Radius (mm)	Repositioning Precision (mm)	Maximum Speed of the Tool (mm/s)
Value	5	850	±0.03	3000

**Table 2:** Main technical parameters of the acceleration sensor and data acquisition instrument.

Acceleration sensor (IEPE: 1A314E)	(1) Sensitivity: 101 mV/g (2) Measuring range: ±500 g (3) Response frequency: 0.5–5000 Hz (X); 0.5–7000 Hz (Y/Z)
Data acquisition instrument (DHDAS: DH5922D)	(1) Number of channels: 8CH + 2CH (2) Maximum analysis bandwidth: DC-50 kHz (3) ADC resolution ratio: 24 bit/channel

**Table 3:** The working conditions of the 40 holes.

Sample Index	Rotation Speed (r/s)	Feeding Speed (mm/s)	Preset Angle
1–5	350	0.5	0°
6–10	350	1	0°
11–15	350	0.5	0.5°
16–20	350	1	0.5°
21–25	350	0.5	1°
26–30	350	1	1°
31–35	350	0.5	1.5°
36–40	350	1	1.5°

**Table 4:** Structure parameters of Resnet.

Layer	Parameter Size	Output Size	Activation
Input	/	1×2000	/
Convolutional	3 × 3, 8	1×1000	/
Residual Unit	$\left[\begin{array}{cc} 3\times 3 & 16\\ 3\times 3 & 16 \end{array}\right]\times 3$	1×500	ReLU
Residual Unit	$\left[\begin{array}{cc} 3\times 3 & 32\\ 3\times 3 & 32 \end{array}\right]\times 4$	1×250	ReLU
Residual Unit	$\left[\begin{array}{cc} 3\times 3 & 64\\ 3\times 3 & 64 \end{array}\right]\times 6$	1×128	ReLU
Residual Unit	$\left[\begin{array}{cc} 3\times 3 & 128\\ 3\times 3 & 128 \end{array}\right]\times 3$	1×64	ReLU
GAP	64	2	Softmax

## Data Availability

The original contributions presented in the study are included in the article, further inquiries can be directed to the corresponding author.
